# Supplementary material for: The Effect of pH and Sodium Caseinate on the Aqueous Solubility, Stability, and Crystallinity of Rutin towards Concentrated Colloidally Stable Particles for the Incorporation into Functional Foods
Source: Molecules. 2022 Jan 14;27(2):534. doi: 10.3390/molecules27020534 (PMC8781550; doi:10.3390/molecules27020534)
Supplement: Supplementary file 1 [file molecules-27-00534-s001.zip › molecules-1492105-supplementary.pdf]

molecules

MDPI

Article

The effect of pH and sodium caseinate on the aqueous solubility, stability, and crystallinity of rutin: towards concentrated colloidally-stable particles for the incorporation into functional foods

Ali Rashidinejad [1,*], Geoffrey B. Jameson [1,2] and Harjinder Singh [1]

[1] Riddet Institute, Massey University, Private Bag 11222, Palmerston North, New Zealand; g.b.jameson@massey.ac.nz (G.B.J.); h.singh@massey.ac.nz (H.S.)
[2] School of Fundamental Sciences, Massey University, 4472, Palmerston North, New Zealand
* Correspondence: A.Rashidinejad@massey.ac.nz

A¶

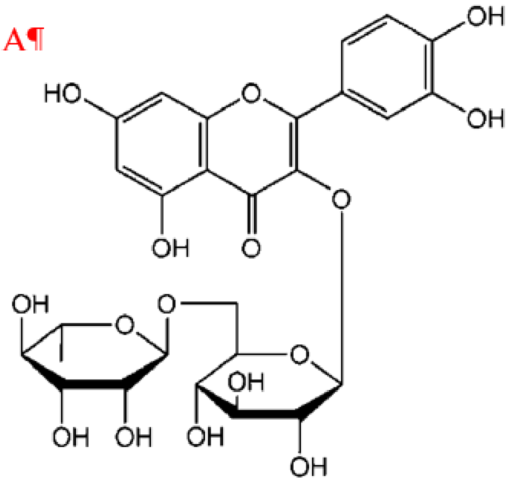

B¶

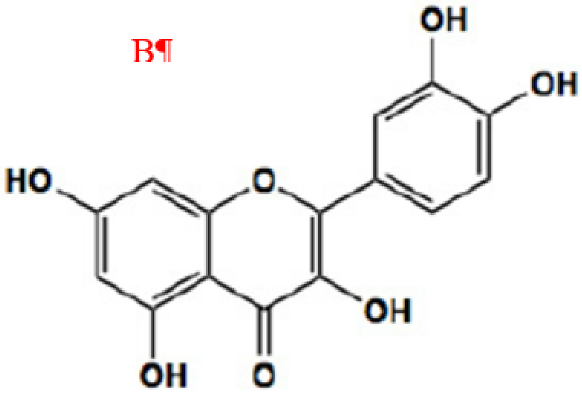

Figure S1. Chemical structure of rutin (A) and quercetin (B).

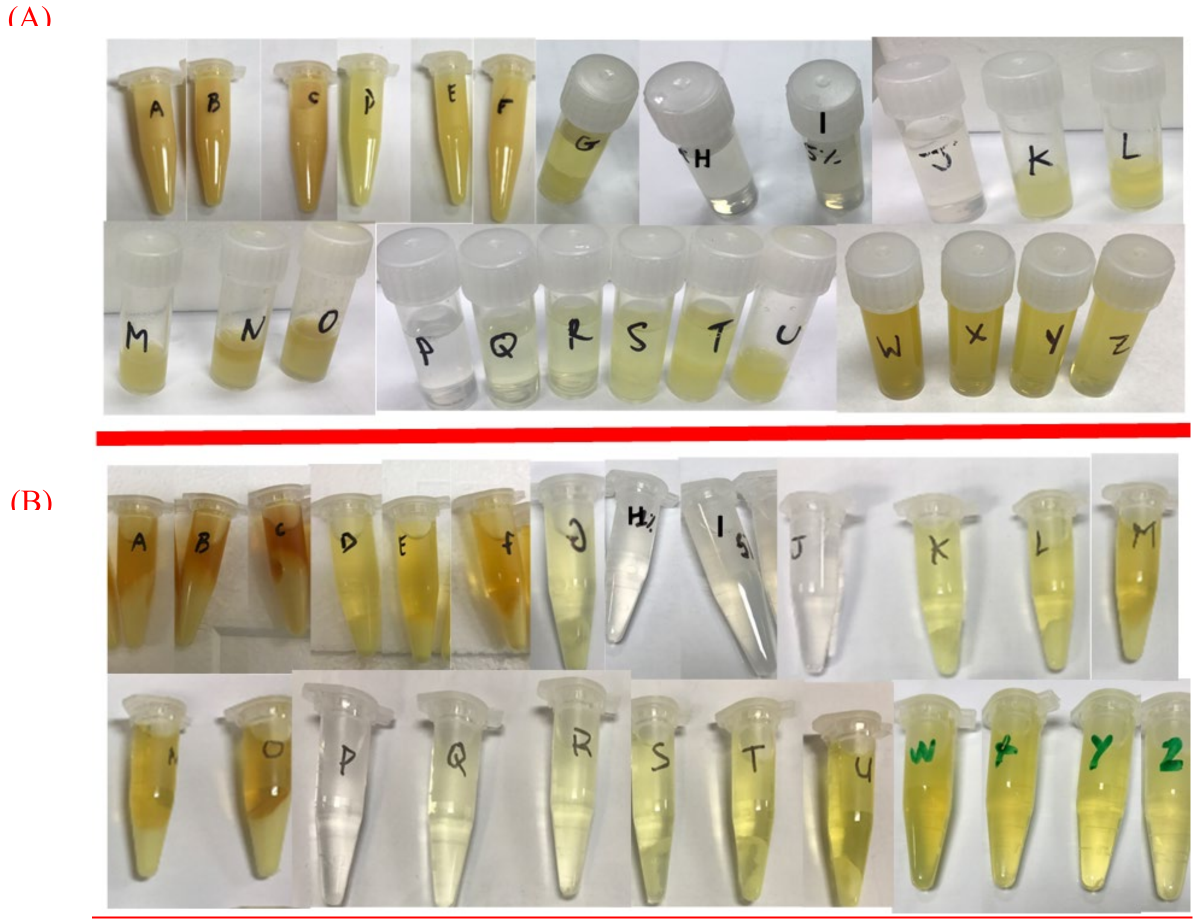

Figure S2. The effect of rutin: NaCas ratio on the physical stability (phase separation) of the formulations before (A) and after (B) centrifugation (3000 × *g*, 10 minutes, 20 °C). For the concentration of protein and rutin, please see Table 1. All the formulations were high-sheer mixed at 33000 rpm for three 1-minute cycles before centrifugation.

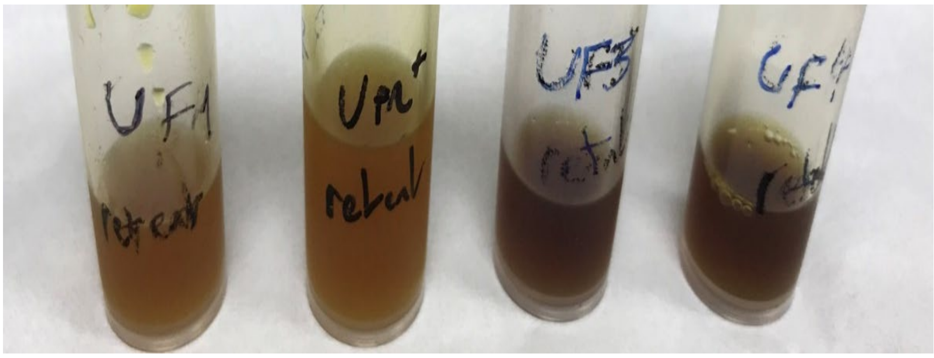

Figure S3. The appearance of the selected encapsulation systems for the delivery of high concentrations of rutin. For the concentration of protein and rutin, please see Table 2.
